# Fatal intracerebral hemorrhage following endovascular Onyx embolization of an arteriovenous malformation: A case report

**DOI:** 10.1097/MD.0000000000036686

**Published:** 2024-01-05

**Authors:** Geo-Seong Park, Jung-Soo Park

**Affiliations:** a Department of Neurosurgery, Jeonbuk National University Hospital, Jeonju, Korea; b Research Institute of Clinical Medicine of Jeonbuk National University, Jeonju, Korea; c Biomedical Research Institute of Jeonbuk National University Hospital, Jeonju, Korea.

**Keywords:** arteriovenous malformation, normal perfusion pressure breakthrough, Onyx embolization, stereotactic radiosurgery

## Abstract

**Introduction:**

Patients with cerebral arteriovenous malformation (AVM) have a lifetime risk of hemorrhagic stroke. Although identified asymptomatic cases can be monitored with imaging follow-up, treatment is considered in cases of AVM rupture or hemodynamic instability.

**Patient concerns:**

A 43-year-old man who had been taking antihypertensive drugs for the past 5 years visited our hospital 3 days after the abrupt onset of headache. The patient also complained of progressive ptosis in the left eye.

**Diagnoses:**

Brain computed tomography (CT) showed a small intraventricular hemorrhage with obstructive hydrocephalus. Subsequent brain CT angiography and magnetic resonance imaging confirmed the presence of an AVM in the cerebellar vermis.

**Interventions:**

Endovascular embolization was performed directly through the right femoral artery. Near-total obliteration of the AVM nidus was achieved by using multiple Onyx castings.

**Outcomes:**

The patient developed an altered mental status and right hemiparesis after the procedure. CT performed after the procedure revealed intraventricular hemorrhage in all ventricles, with a left thalamic intracerebral hemorrhage. Despite emergency external ventricular drainage and aggressive treatment for intracranial pressure control, the patient expired on the 14th day after the embolization procedure.

**Lessons:**

When treating AVMs, especially those with a large nidus of high flow, it is necessary to consider possible hemorrhagic complications and preventive measures.

## 1. Introduction

Although the incidence of brain arteriovenous malformation (AVM) remains incompletely defined, according to population-based studies, the overall prevalence of brain AVM ranges from 1.10 to 1.42 cases per 100,000.^[1]^ Intracranial hemorrhage is the most common and devastating symptomatic manifestation of brain AVMs, with permanent neurological morbidity and mortality occurring in approximately 7% of cases.^[2]^ The annual rupture risk of untreated AVMs is estimated to be 1% to 4.61%.^[1,3]^ Although identified asymptomatic cases can be monitored with imaging follow-up, treatment is considered in cases of AVM rupture or hemodynamic instability.

With the improvement of embolization materials and endovascular techniques, endovascular treatment is being increasingly used in the multimodal management of brain AVMs. Endovascular embolization is mainly used for nidus reduction before microsurgery or radiosurgery and palliative treatment; however, in some cases it is used for curative intents.^[1,4,5]^

We describe a case of a fatal intracranial hemorrhage following endovascular Onyx embolization of a brain AVM and discuss AVM treatment considerations.

## 2. Case report

**Figure 1.**
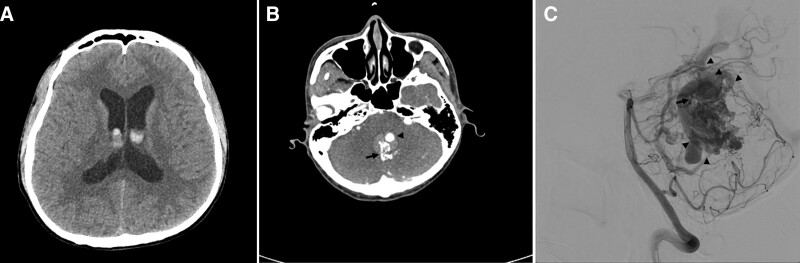
(A) Non-enhanced head computed tomography (CT) demonstrated a small intraventricular hemorrhage with enlarged ventricle. (B) CT angiography revealed an arteriovenous malformation (AVM) nidus (arrow) with dilated draining veins in the cerebellum (arrow head). (C) Lateral projection of the left vertebral artery distal subtraction angiography. The nidus was approximately 3.2 cm in maximum diameter, mainly supplied by the engorged branch of left superior cerebellar artery (SCA) (arrow). Two main dilated venous outlets were observed draining into the vein of Labbé, internal cerebral vein, and vein of Galen with aneurysmal dilatation (arrow head).

A 43-year-old man with a 5-year history of taking hypertension medication presented to our emergency department 3 days after an abrupt onset of headache. The patient also complained of progressive ptosis in the left eye. All laboratory tests and electrocardiograms were within the normal ranges. Non-enhanced head computed tomography (CT) demonstrated a small intraventricular hemorrhage with an enlarged ventricle (Fig. 1A). Subsequent CT angiography revealed an AVM nidus with dilated draining veins in the cerebellum (Fig. 1B). As the ptosis was judged to be a sunset sign caused by progressive hydrocephalus, the patient was admitted to the intensive care unit after external ventricular drainage. Digital subtraction angiography performed 2 days after hospitalization revealed an AVM of the cerebellar vermis with a high-flow shunt. The nidus was approximately 3.2 cm in maximum diameter and was mainly supplied by the engorged branch of the left superior cerebellar artery. Two main dilated venous outlets were observed draining into the vein of Labbé, internal cerebral vein, and vein of Galen, with aneurysmal dilatation (Fig. 1C). The AVM was Spetzler–Martin grade III (nidus size, 3–6 cm: 1; deep venous drainage: 1; non-eloquent location: 0).

**Figure 2.**
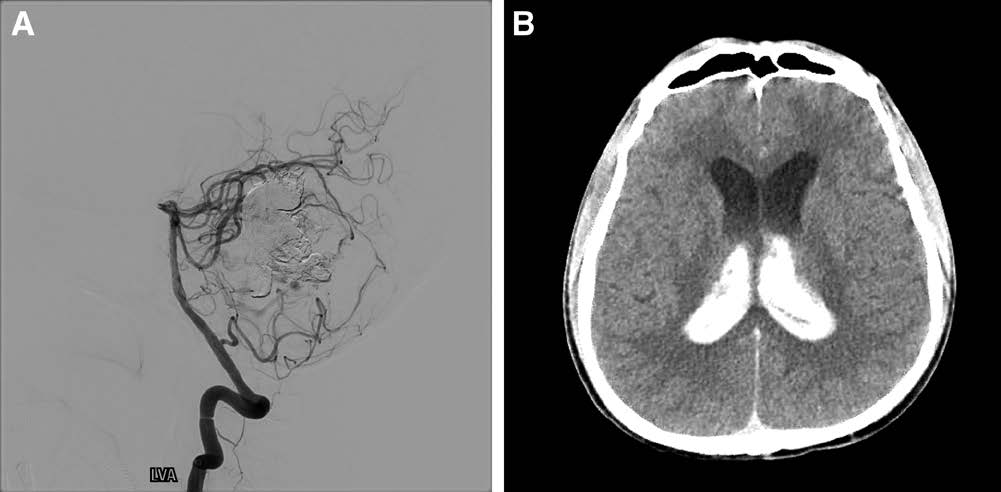
(A) Postprocedural angiogram of the left vertebral artery showed near total obliteration of the arteriovenous malformation (AVM) nidus. (B) Postprocedural head computed tomography (CT) revealed an interventricular hemorrhage in all ventricles with obstructive hydrocephalus.

After discussing with the neurosurgeon and neurointerventionist, we decided to perform endovascular treatment because of the high surgical risk. The procedure was performed under general anesthesia via the femoral artery access. First, a 5-Fr SOFIA intermediate catheter (MicroVention, Tustin, CA) was inserted into the midportion of the basilar artery. A Marathon microcatheter (Medtronic, Irvine, CA) was then navigated into the left superior cerebellar artery and wedged into the AVM nidus using a Mirage microguidewire (Medtronic, Irvine, CA). After confirming the position of the microcatheter using superselective angiography, the liquid embolic agent Onyx (Medtronic, Irvine, CA) was injected slowly under fluoroscopic guidance. The main portion of the AVM nidus and dilated draining vein were obliterated with the injection of 3.5 mL of Onyx (Fig. 2A). At the end of the procedure, the patient’s blood pressure suddenly increased to 180 mm Hg; therefore, the procedure was stopped and CT was performed immediately. Head CT revealed interventricular hemorrhage in all ventricles, with intracerebral hemorrhage in the left thalamus (Fig. 2B). Despite emergent external ventricular drainage and aggressive treatment to lower the intracranial pressure, the patient’s mental status deteriorated. Subsequently, an additional multifocal intracerebral hemorrhage occurred, and the patient eventually died on the 14th day after the procedure.

## 3. Discussion

Brain AVMs are rare cerebrovascular diseases characterized by a nidus, feeding arteries, and draining veins. With the increasingly widespread use of noninvasive neuroimaging techniques such as magnetic resonance imaging, incidental AVMs are being detected with greater frequency. According to a Randomized Trial of Unruptured Brain AVMs (ARUBA), conservative management was superior to interventional therapy for the prevention of death or stroke in patients with unruptured brain AVMs.^[6]^ However, the methodology and findings of the ARUBA trial have been contentious owing to the follow-up duration, heterogeneity of treatment modalities, and higher-than-expected primary endpoints and hemorrhage rates in the intervention arm compared with those of previous observational studies.^[1]^ Therefore, treatment decisions for patients with brain AVMs are based on natural-course risk estimates weighed against outcome data from invasive interventions.^[7]^

Intracranial hemorrhage is the most common symptomatic manifestation of a brain AVM, and the overall hemorrhagic risk of untreated, unruptured AVMs is estimated to be 1% to 3% per year.^[3,8]^ Prior hemorrhage is, however, the most consistent predictor of subsequent hemorrhage. Interventions, such as microsurgery, endovascular treatment, and stereotactic radiosurgery, are considered in patients with ruptured AVMs. Microsurgery has the advantages of a high rate of complete obliteration, immediate elimination of hemorrhagic risk, and long-term durability compared with the other AVM interventions; however, it has the disadvantages of a long recovery period, higher perioperative morbidity rate, and difficulty in accessing AVMs located in eloquent brain regions.^[9]^ Stereotactic radiosurgery can be a suitable treatment option for lesions in deep or eloquent brain regions that are difficult to access surgically or small-sized AVMs; however, it takes a long time to observe the effect of obliteration and the risk of bleeding persists during the latency period.^[10]^

Endovascular treatment is frequently used in multimodal management of AVMs, such as nidus reduction before surgery or radiosurgery, curative embolization, and palliative embolization,^[1,5]^ and can be used to treat brain AVMs in locations where surgical access is difficult. As various embolization materials have been developed, including polyvinyl alcohol foam particles, platinum coils, liquid n-butyl cyanoacrylate, and ethylene vinyl alcohol (Onyx), the frequency of endovascular treatment is increasing.^[11]^ Onyx is a nonadhesive agent with controllable endovascular behavior that allows more precise nidus penetration to create a solid cast, and thus, it has been widely used in the treatment of brain AVMs. However, unexpected periprocedural hemorrhages have been associated with endovascular treatment, and previous studies have suggested several causes, such as vessel perforation by the device, catheter adhesion, drain vein occlusion, and changes in pressure loading.^[9,12]^ In particular, the cause of hemorrhage after a successfully completed procedure may be explained by the normal perfusion pressure breakthrough (NPPB) phenomenon. The brain adjacent to the AVM is subjected to long-standing vascular steal and may lose its normal vascular autoregulation. After embolization of the AVM, hyperperfusion may occur in the surrounding brain parenchyma, potentially causing cerebral edema and hemorrhage.^[4,8,12,13]^ The risk factors for the development of NPPB include supratentorial location, lobar topography, high-flow AVM, large and compact nidus, multiple feeding arteries, deep venous drainage, high amounts of injection materials, and large AVMs with embolization performed in a single session.^[12,14]^ In our case, it is highly likely that NPPB occurred while trying to treat the high-flow and large AVM in a single embolization session.

Although endovascular treatment can be used for various purposes in the treatment of brain AVMs, neurointerventionists should be aware of the periprocedural hemorrhagic complications. It is necessary to predict the development of NPPB after treatment through meticulous angiography and perfusion studies, such as perfusion CT, magnetic resonance imaging, or single-photon emission CT. In particular, when endovascular treatment of a large, high-flow AVM is chosen, staged embolization should be considered, even if the procedure proceeds smoothly without any problems.

## Author contributions

**Writing – original draft:** Geo-Seong Park.

**Writing – review & editing:** Jung-Soo Park.
